# The impact of elective total hip and knee arthroplasty on physical performance in orthogeriatric patients: a prospective intervention study

**DOI:** 10.1186/s12877-023-04460-6

**Published:** 2023-11-21

**Authors:** Tobias Kappenschneider, Philip Bammert, Günther Maderbacher, Felix Greimel, Dominik Emanuel Holzapfel, Timo Schwarz, Julia Götz, Stefano Pagano, Markus Scharf, Katrin Michalk, Joachim Grifka, Matthias Meyer

**Affiliations:** 1 Department of Orthopaedic Surgery, Regensburg University Medical Center, Bad Abbach, Germany; 2 Department of Health Economics, Technical University of Munich, Munich, Germany

**Keywords:** Orthogeriatric, SPPB, Hip arthroplasty, Knee arthroplasty, Older people

## Abstract

**Background:**

Osteoarthritis is a prevalent condition in older adults that leads to reduced physical function in many patients and ultimately requires hip or knee replacement. The aim of the study was to determine the impact of hip and knee arthroplasty on the physical performance of orthogeriatric patients with osteoarthritis.

**Methods:**

In this prospective study, we used data from 135 participants of the ongoing Special Orthopaedic Geriatrics (SOG) trial, funded by the German Federal Joint Committee (GBA). Physical function, measured by the Short Physical Performance Battery (SPPB), was assessed preoperatively, 3 and 7 days postoperatively, 4–6 weeks and 3 months after hip and knee arthroplasty. For the statistical analysis, the Friedman test and post-hoc tests were used.

**Results:**

Of the 135 participants with a mean age of 78.5 ± 4.6 years, 81 underwent total hip arthroplasty and 54 total knee arthroplasty. In the total population, SPPB improved by a median of 2 points 3 months after joint replacement (p < 0.001). In the hip replacement group, SPPB increased by a median of 2 points 3 months after surgery (p < 0.001). At 3 months postoperatively, the SPPB increased by a median of 1 point in the knee replacement group (p = 0.003).

**Conclusion:**

Elective total hip and knee arthroplasty leads to a clinically meaningful improvement in physical performance in orthogeriatric patients with osteoarthritis after only a few weeks.

**Trial registration:**

This study is part of the Special Orthopaedic Geriatrics (SOG) trial, German Clinical Trials Register DRKS00024102. Registered on 19 January 2021.

**Supplementary Information:**

The online version contains supplementary material available at 10.1186/s12877-023-04460-6.

## Background

Osteoarthritis (OA) is considered the most prevalent chronic joint disease in the world and one of the most common causes of pain and disability in older people [1]. About half of the world’s population aged 65 years and older is affected by OA [2]. 80% of people with symptomatic OA have limited mobility, while 25% are unable to carry out normal daily activities [3, 4]. Several studies have also found an association between OA and frailty [5, 6]. Reduced mobility is part of the frailty syndrome. With the increasing incidence of OA, more and more older people are facing severe financial and social burdens [3]. Therefore, prevention and treatment of OA must be a high priority in health and socioeconomic policy.

The prevalence of osteoarthritis increases with age because the disease is not reversible. OA of the hip and knee is a major cause of impaired physical performance and disability, especially in older people [3]. Increasing symptoms and loss of joint function often lead to hip or knee replacement surgery. Due to demographic trends, the number of primary hip and knee arthroplasties will increase dramatically in the coming years. For example, the number of elective total hip and total knee arthroplasties (THA and TKA) in the US is expected to increase by 71% and 85% respectively by 2030 [7].

The aim of surgical joint replacement is to restore joint function and thus reduce pain and improve mobility as well as restore social participation according to the World Health Organization (WHO) model of disability. There is a large body of literature on functional recovery after hip and knee replacement, but it is often based on younger patients and uses the Timed Up and Go Test (TUG) to assess mobility [8, 9]. However, little is known about the increase in physical performance in orthogeriatric patients after primary total hip and knee arthroplasty as measured by the Short Physical Performance Battery (SPPB). It is a commonly used, validated test of physical function in geriatrics. SPPB evaluates balance, mobility and muscle strength by examining an individual’s ability to stand in different positions, time to walk 4 m, and time to rise up from and sit down on a chair 5 times [10]. So far, only a pilot study with four patients has investigated the feasibility and acceptability of the SPPB as a clinically applicable, simple and objective test to assess physical function in older adults after joint replacement [11].

In Germany, total hip and knee arthroplasty are among the 20 most common surgical procedures for hospitalised patients overall [12]. The aim of the study was to determine the impact of elective total hip and knee arthroplasty on the physical performance of older adults with OA. We hypothesised that hip and knee joint replacement in orthogeriatric patients would be associated with an improvement in physical performance as measured by the SPPB.

## Methods

### Study design

This study is part of the ongoing Special Orthopaedic Geriatrics (SOG) trial (German Clinical Trials Register, 19/01/2021, DRKS00024102). The SOG study is a monocentric, prospective, randomised controlled trial funded by the German Federal Joint Committee (GBA). The original study aimed to investigate a specially developed multimodal care model (SOG care model) for orthogeriatric patients with total hip and total knee arthroplasty compared to usual orthopaedic care without orthogeriatric co-management. Physical performance as measured by the SPPB was the primary outcome measure. A detailed description of the study can be found elsewhere [13]. The current study enrolled 145 patients from the SOG trial between 01April 2021 and 15 April 2023. This additional analysis was not planned when the original study was designed.

### Data collection

In the Orthopaedics Department of our University Centre, about 18,000 patients are treated annually in the university outpatient clinic and > 1,500 endoprosthetic procedures on hip and knee joints are performed each year. Participants were recruited at the university outpatient clinic if they were diagnosed with primary hip or knee osteoarthritis and had an indication for THA or TKA. The study data were collected preoperatively (t1), 3 and 7 days postoperatively (t2 and t3), 4–6 weeks (t4) and 3 months (t5) after surgery.

### Study population

Eligibility criteria included: primary hip or knee osteoarthritis, age ≥ 70 years and multimorbidity or age ≥ 80 years and indication for elective unilateral hip or knee replacement. Exclusion criteria were age < 70 years, previous bony surgery or tumour in the area of the joint to be treated, acute infection and increased need for care (care level ≥ 4; severe impairment of independence, need for help with basic care 24 h a day).

Out of a total of 145 subjects in the SOG study, there were 10 drop-outs. The reasons were cancellation of surgery or refusal to participate in the study. As a result, the number of people included in the analysis was 135. In 5 participants, SPPB could not be performed on postoperative days 3 and 7. The number of patients lost to follow-up was 8 at 4–6 weeks (follow-up 1) and 8 at 3 months (follow-up 2).

### Surgical techniques and implants

All operations were performed in a single Department of Orthopaedic Surgery of a University Medical Centre. The lateral decubitus position was used for the THA. A minimally invasive anterolateral approach was chosen [14]. Press-fit acetabular components and stems from a single manufacturer (Pinnacle cup, Corail stem; DePuy, Warsaw, IN) were used in all THAs. Cementless stems were preferred. Knee arthroplasty was performed via a medial parapatellar approach. Cemented components from a single manufacturer (PFC Sigma; DePuy) were used in all TKAs. Patella resurfacing was not performed. All patients were mobilised under full weight bearing immediately after surgery. Postoperatively, patients with THA and TKA remained in hospital for 7 days unless complications occurred. They received daily physiotherapy and were then discharged to inpatient rehabilitation. With a few exceptions, outpatient rehabilitation was performed at the patient’s request. Most patients were transferred to the rehabilitation clinic belonging to the hospital. The duration of inpatient and outpatient rehabilitation was three weeks.

### Assessment of physical performance

Physical performance was measured using the Short Physical Performance Battery (SPPB), a commonly used and validated assessment in geriatrics that includes three objective tests of lower body function [8]. SPPB evaluates balance, mobility and muscle strength by examining an individual’s ability to stand in different positions, time to walk 4 m, and time to rise up from and sit down on a chair 5 times. The individual tests are scored between 0 and 4, with a maximum total score of 12 (range 0–12). Higher total scores indicate better lower body function [10, 15]. Small meaningful changes in the SPPB are present at 0.5 points, substantial changes are assumed from a 1-point improvement [16].

### Statistical analysis

Descriptive statistics including demographic and morbidity-related characteristics were calculated for the whole sample. As a core statistical method, the non-parametric Friedman test was employed, as an alternative to the one-way repeated measures ANOVA. The Friedman test was preferred due to the scale and distribution of the outcome variable. It was tested whether there were significant differences between the five times of measurement, with the SPPB score being the dependent variable of interest. Medians and interquartile ranges were reported for each time of measurement alongside the respective *p*-values yielded by the Friedman test. Post-hoc tests were then performed for significant differences comparing all of the time points against each other using Wilcoxon signed-rank tests. In this procedure Bonferroni-correction was applied to reduce the risk of a type I error. Effect sizes and *p*-values will be reported for each comparison. Further analyses included Friedman tests as well as post-hoc tests for hip replacement surgery patients and knee replacement surgery patients separately as well as for each of the three subscores (standing balance, 4 m gait speed test, and the timed five-repetition sit-to-stand test) of the SPPB. Taking into account the drop-outs and patients lost to follow-up described under study population, the following number of patients were available for the analyses: t1: n = 135, t2: n = 130, t3: n = 130, t4: n = 127, t5: n = 127. All analyses were conducted in R version 4.2.1. using the package using the R-package “rstatix”. *p*-values p < 0.05 were considered statistically significant.

## Results

Out of a total of 135 participants, 81 patients underwent total hip arthroplasty and 54 patients underwent total knee arthroplasty. The female gender was represented more frequently overall (65.2%). The mean age of all participants was 78.5 ± 4.7 years, in the hip replacement group it was 78.1 ± 4.5 years and in the knee replacement group 79.1 ± 4.8 years. All study participants had a mean of 7.6 ± 2.9 comorbidities. The mean Charlson Comorbidity Index (CCI) for the total population was 5.3 ± 1.8 and the overall mean PFP (Physical Frailty Phenotype) score was 2.1 ± 1.3. (Table 1).

**Table 1:** Baseline characteristics of the 135 participants

Characteristics	Total (n = 135)	Hip arthroplasty (n = 81)	Knee arthroplasty (n = 54)
Female n (%)	88 (65.2)	51 (63.0)	37 (68.5)
Age y, mean ± SD	78.5 ± 4.7	78.1 ± 4.5	79.1 ± 4.8
BMI kg/m² mean ± SD	28.8 ± 4.9	28.6 ± 5.4	29.2 ± 4.1
Comorbidities n mean ± SD	7.6 ± 2.9	7.3 ± 3.0	8.0 ± 2.8
CCI mean ± SD	5.3 ± 1.8	5.3 ± 2.0	5.4 ± 1.5
Barthel Index (0-100) mean ± SD	92.4 ± 12.3	92.2 ± 12.0	92.8 ± 12.8
IADL score (0–8) mean ± SD	6.7 ± 1.6	6.5 ± 1.6	6.9 ± 1.4
PFP score (0–5) mean ± SD	2.1 ± 1.3	2.5 ± 1.2	1.7 ± 1.3
MMSE score (0–30) mean ± SD	26.8 ± 2.7	27.0 ± 2.8	26.7 ± 2.5
GDS-15 score (0–15) mean ± SD	3.3 ± 2.9	3.4 ± 2.8	3.1 ± 3.1

SD, Standard deviation; BMI, Body Mass Index; CCI, Charlson Comorbidity Index; NRS, Nutritional Risk Screening; IADL, Instrumental Activities of Daily Living; PFP, Physical Frailty Phenotype (Fried); MMSE, Mini-Mental State Examination; GDS, Geriatric Depression Scale

Using the Friedman test, we first examined whether there were significant differences between the five measurement points of the SPPB score. In the total sample as well as in the hip and knee arthroplasty groups, there were significant differences (p < 0.001) between at least two of the SPPB values measured perioperatively at 5 different time points (Table 2).

**Table 2:** Differences in SPPB score by group between each time of measurement, Md (IQR)/Friedman

	Pre-op	Post-op d3	Post-op d7	Post-op 4–6 wk	Post-op 3 mo	p
SPPB total	7 (5–9)	4 (3–6)	7 (5–8)	8 (6–10)	9 (7–11)	**< 0.001**
SPPB hip	7 (4–9)	5 (3–6)	7 (5–9)	9 (6–10)	9 (7–11)	**< 0.001**
SPPB knee	8 (6–9)	4 (2–6)	6 (5–8)	8 (6–9)	9 (6–11)	**< 0.001**

Md, Median; IQR, Interquartile Range; SPPB, Short Physical Performance Battery; p, *p*-value of the Friedman test; d, day; wk, week; mo, months

### Total study group

**Fig. 1:**
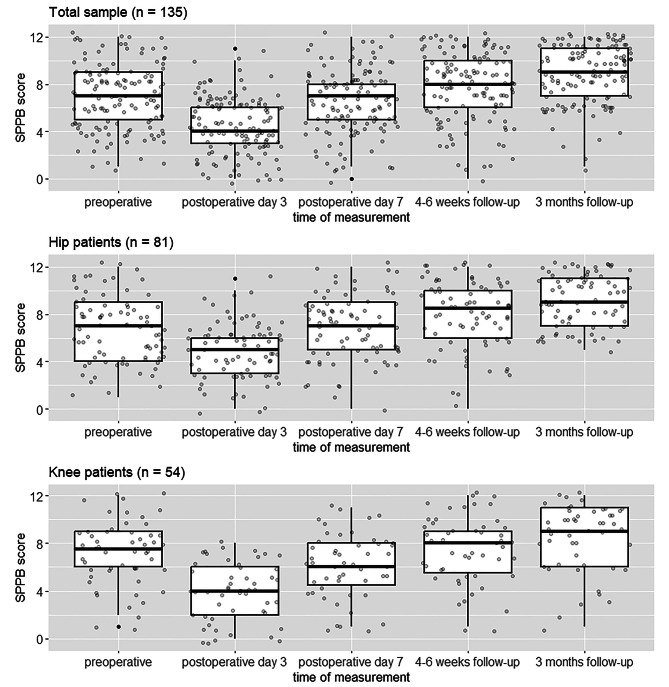
SPPB scores of the total study group, the hip and knee replacement groups at the 5 different measurement points (Grey dots indicate individual observations; Black dots indicate outliers)

Patients had intermediate to low physical performance preoperatively with a median SPPB score of 7 (IQR 5–9). On postoperative day 3, there was a significant decrease in the SPPB score to a median of 4 (p < 0.001). After three months, the median SPPB in the total population improved by 2 points (p < 0.001) compared to the baseline median (Fig. 1; Table 3).

### Hip arthroplasty group

The hip patients had already returned to their pre-surgery baseline on postoperative day 7 (hospital discharge) with a median score of 7. After 4–6 weeks, the SPPB even increased by 2 points (from a baseline value of 7 to a value of 9) (p = 0.005). After 3 months, the median SPPB remained 9 (Tables 2 and 3).

### Knee arthroplasty group

The decrease in SPPB was particularly pronounced in the knee arthroplasty group, from a median score of 8 preoperatively to a median of 4 postoperatively (p < 0.001). In this cohort, the baseline SPPB was not reached with a median value of 6 on day 7 after surgery. This was only the case 4–6 weeks after surgery. After 3 months, the median SPPB in the knee group improved by 1 point (p = 0.003) compared to the baseline median (Fig. 1; Table 3).

**Table 3:** Post-hoc test for each comparison of the Short Physical Performance Battery (SPPB) measurement time points (t1-t5)

**SPPB total sample**					
**contrast**	**t1-t2**	**t1-t3**	**t1-t4**	**t1-t5**	**t2-t3**
p*	**< 0.001**	0.763	**0.034**	**< 0.001**	**< 0.001**
r	0.659	0.155	0.226	0.699	0.742
**contrast**	**t2-t4**	**t2-t5**	**t3-t4**	**t3-t5**	**t4-t5**
p*	**< 0.001**	**< 0.001**	**< 0.001**	**< 0.001**	**< 0.001**
r	0.783	0.863	0.498	0.789	0.569
**SPPB hip replacement group**					
**contrast**	**t1-t2**	**t1-t3**	**t1-t4**	**t1-t5**	**t2-t3**
P*	**< 0.001**	1	**0.005**	**< 0.001**	**< 0.001**
r	0.543	0.040	0.384	0.786	0.737
**contrast**	**t2-t4**	**t2-t5**	**t3-t4**	**t3-t5**	**t4-t5**
P*	**< 0.001**	**< 0.001**	**< 0.001**	**< 0.001**	**< 0.001**
r	0.754	0.866	0.452	0.778	0.533
**SPPB knee replacement group**					
**contrast**	**t1-t2**	**t1-t3**	**t1-t4**	**t1-t5**	**t2-t3**
P*	**< 0.001**	**0.009**	1	**0.003**	**< 0.001**
r	0.796	0.458	0.063	0.527	0.755
**contrast**	**t2-t4**	**t2-t5**	**t3-t4**	**t3-t5**	**t4-t5**
P*	**< 0.001**	**< 0.001**	**< 0.001**	**< 0.001**	**< 0.001**
r	0.833	0.862	0.570	0.810	0.632

t1 = preoperative, t2 = 3. postoperative day, t3 = 7. postoperative day, t4 = 4–6 weeks postoperative, t5 = 3 months postoperative

*Bonferroni correction applied for 10 tests; p = *p*-value of the Pairwise Wilcoxon signed-rank Test; r = effect size

**Fig. 2:**
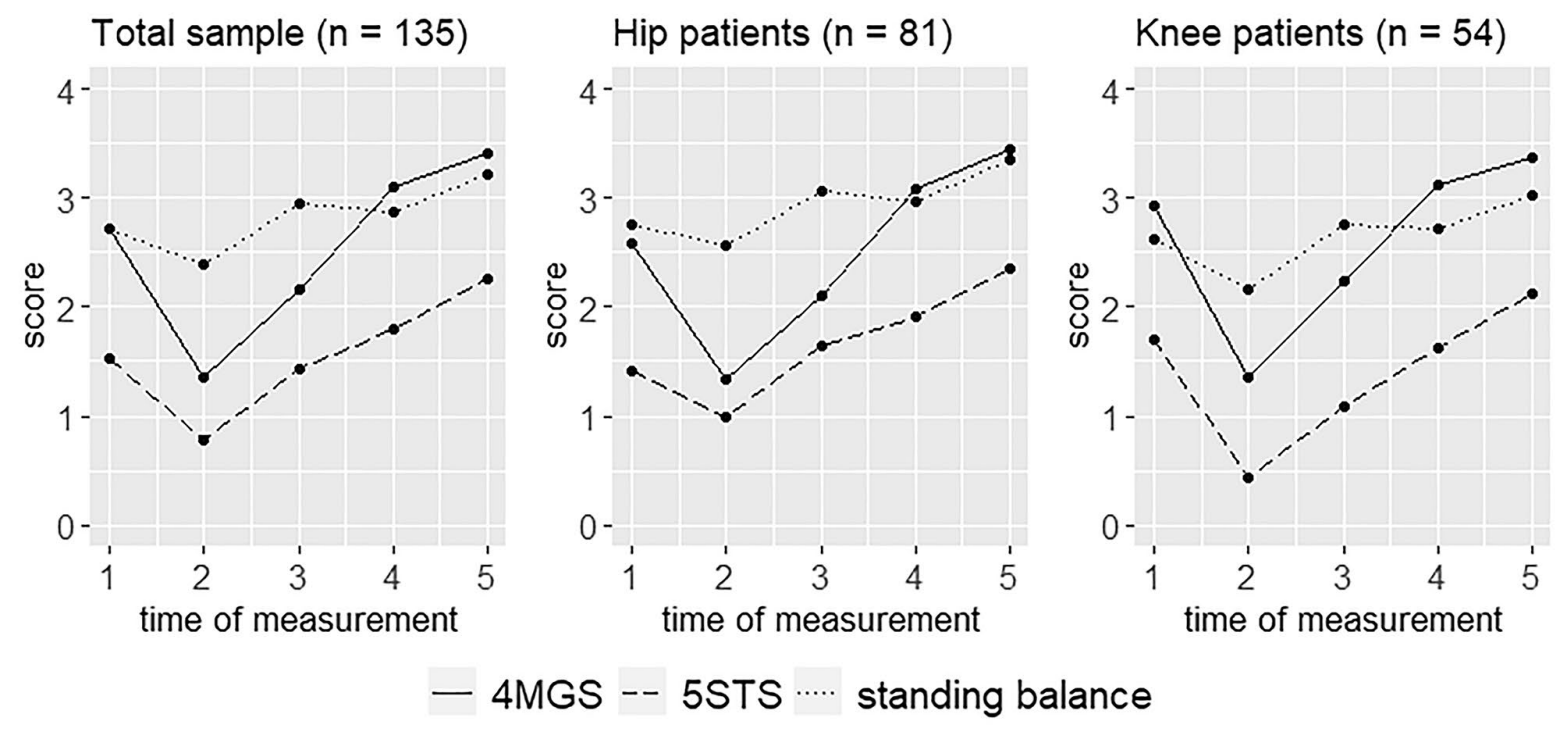
Differences in the SPPB subscores standing balance, 4-meter gait speed test (4MGS) and 5 times sit to stand test (5STS) at the different measurement time points (t1 = preoperative, t2 = postoperative day 3, t3 = postoperative day 7, t4 = 4–6 weeks postoperative, t5 = 3 months postoperative)

Figure 2 describes the differences in the SPPB subscores standing balance, 4-meter gait speed test (4MGS) and 5 times sit to stand test (5STS) between the five different measurement points (t1-t5). The Friedman test showed significant differences in all three subscores between the five measurement points (Supplementary Table 1). With the exception of the knee replacement group in the balance subscore, all other groups improved by a median of one point between preoperative assessment and measurement 3 months after surgery.

## Discussion

In our study, SPPB increased by 2 points 3 months after THA and by 1 point 3 months after TKA. Perera et al. considered a change in the SPPB total score of 0.5 points as a small significant change. A change in the SPPB of 1.0 points or more is assumed to be a substantial change [16]. This means that after both elective THA and TKA, there is a substantial improvement in the physical performance of orthogeriatric patients with OA 3 months after surgery. In line with the results of Perera et al. [16], the SPPB score decreased in the first days after surgery, but returned to baseline by postoperative day 7 in the total population (hospital discharge). The THA group demonstrated substantial improvement in SPPB 4–6 weeks postoperatively (discharge from rehabilitation). The TKA group showed substantial improvement in SPPB 3 months after surgery.

To assess physical performance, the SPPB is commonly used in geriatrics in both clinical and research contexts [10]. Przkora et al. evaluated the applicability and acceptability of SPPB before and after TKA in 2021 [11]. The results indicated that SPPB is easy to perform and can be easily integrated into daily clinical practice. Although the study only investigated SPPB in knee replacement, Przkora et al. concluded that assessing SPPB in the perioperative setting in patients undergoing lower extremity joint replacement surgery is feasible and acceptable [11]. The SPPB is therefore an objective test for the simple assessment of physical function in older adults undergoing elective TKA and THA with known high reliability, validity and responsiveness. As a measure of postoperative functional outcome, the SPPB has mainly been studied and used in patients undergoing cardiac or pulmonary surgery [17, 18].

SPPB is correlated with sarcopenia, frailty, disability and mortality [15, 19–21]. It is possible that all these factors can be improved after THA and TKA. OA of the hip and knee is a significant barrier to physical activity and is one of the top five causes of disability in American adults [22]. According to Guralnik et al., increasing physical function can lead to the prevention of severe, especially mobility-related, disabilities on the one hand, and promote recovery from disabilities on the other [15]. A 1-point improvement in the SPPB summary score has been indicated as clinically meaningful and correlated well with increases in overall activity and survival [15]. In their review, de Fátima Ribeiro Silva et al. reported numerous studies in which lower SPPB scores (range 0–6 points) were associated with a significantly increased risk of death [23]. In addition, SPPB is related to activities of daily living (ADLs), falls, dyspnoea, postoperative complications, cardiovascular disease, institutionalisations and hospitalisations [23].

Compared to non-surgical interventions, THA and TKA seems superior in improving physical performance in older patients with OA and multimorbidity. In a cluster randomised trial comparing two community-based programmes, Zgibor et al. showed an improvement in SPPB of 0.3 and 0.5 points respectively after 6 months [24]. In our study, participants’ SPPB scores improved by a median of 2.0 points at three months after surgical hip and knee replacement compared to preoperative baseline. The participants in the study by Zgibor et al. were younger (included age ≥ 50 years, mean age 72.7 ± 7,8 years). Also, arthritis was not defined more precisely and no severity was given, so mild forms of arthritis may have been included [24].

An alternative test often used in studies to assess mobility is the Timed Up and Go Test (TUG) [25]. Compared to the SPPB [10], it is faster and allows the use of armrests. However, it always requires the patient to stand up. As long as standing up is not possible independently, progress in walking cannot be shown. The test is therefore susceptible to floor effects. In contrast, the SPPB is less vulnerable as a test battery [10]. The SPPB is a widely studied and well-validated tool with good test quality criteria and particularly good reliability. Studies identified good prognostic properties for functional deterioration, mortality, institutionalisations and duration of clinical treatments [10, 15, 19–21, 23]. Unnanuntana et al. and also Pongcharoen et al. demonstrated significantly shorter TUG test times after TKA at 3 months in their 2018 and 2023 studies [9, 26]. Although these studies were not conducted in orthogeriatric patients, their results are consistent with our demonstrated improvements in mobility with SPPB.

This study has some limitations. The study is part of the ongoing SOG trial and the analyses presented here were not originally planned. For this reason, there is no control group for this evaluation. Although the participants had exhausted all conservative measures (analgesics including opioids, physiotherapy and often rehabilitation) as a prerequisite for surgery, there are always circumstances that could have influenced physical performance even without special intervention. This may include, for example, psychosocial aspects or the treatment of comorbidities. The improvement in physical performance is not exclusively a benefit of the surgery. Post-operative care by the healthcare team, physiotherapy and rehabilitation for functional exercise after joint replacement also contribute. The results were observed in this context. The additional impact of orthogeriatric co-management is not clear, especially as such care models can be very heterogeneous. Prestmo et al. observed no significant difference between orthopaedic treatment and orthogeriatric co-management in the context of hip fractures in both SPPB and TUG within 1 month after surgery. Only at 4 months and 1 year did significant differences appear for SPPB, but not for TUG [27].

A major strength of this study is its prospective design with 135 participants. Apart from the pilot study by Przkora et al. with a total of 4 subjects [11], we are not aware of any other study on this issue. The mean age of the total population of this study is 78.5 ± 4.7 years. Many geriatric studies also accept significantly younger participants, sometimes from the age of 50. Furthermore, these are not only very old patients, but also multimorbid and often pre-frail/frail participants. The close collaboration between orthopaedists and geriatricians in the SOG study made it possible for the first time to address this issue using an established and validated geriatric assessment tool such as the SPPB. Although many participants had OA in other joints, which worsened in some patients in the further course after surgery, a considerable improvement in physical performance could still be achieved through THA or TKA. Data analysis was performed externally and independently by the Department of Health Economics at the Technical University of Munich.

## Conclusion

Elective total hip and knee arthroplasty leads to a clinically meaningful improvement in physical performance in orthogeriatric patients with OA. With total hip replacements, there is a substantial increase in SPPB as early as 4–6 weeks after surgery. Knee replacement demonstrates substantial improvement in SBBP 3 months after surgery.

### Electronic supplementary material

Below is the link to the electronic supplementary material.

Supplementary Material 1

Supplementary Material 2

## Data Availability

The data are available on reasonable request from the corresponding author.

## Abbreviations

BMI: Body Mass Index
CCI: Charlson Comorbidity Index
d: Day
GDS: Geriatric Depression Scale
IADL: Instrumental Activities of Daily Living
IQR: Interquartile Range
Md: Median
MMSE: Mini-Mental State Examination
mo: months
NRS: Nutritional Risk Screening
OA: Osteoarthritis
p: *p*-value
PFP: Physical Frailty Phenotype (Fried)
SD: Standard deviation
SOG: Special Orthopaedic Geriatrics
SPPB: Short Physical Performance Battery
THA: Total hip arthroplasty
TKA: Total knee arthroplasty
TUG: Timed Up and Go Test
w: week
WHO: World Health Organization
