# Supplementary material for: The impact of elective total hip and knee arthroplasty on physical performance in orthogeriatric patients: a prospective intervention study
Source: BMC Geriatr. 2023 Nov 21;23:763. doi: 10.1186/s12877-023-04460-6 (PMC10664286; doi:10.1186/s12877-023-04460-6)
Supplement: Supplementary file 1 — Supplementary Material 1 [file 12877_2023_4460_MOESM1_ESM.pdf]

Additional file 1

Supplementary Table 1 Differences in SPPB subscores balance, gait speed and chair rise, Md (IQR)/Friedman test

SPPB Subscore	Pre-op	Post-op d3	Post-op d7	Post-op 4-6 wk	Post-op 12 wk	p
Balance total	3 (2-4)	2 (2-4)	3 (2-4)	3 (2-4)	4 (3-4)	**<0.001**
Balance hip	3 (2-4)	2 (2-4)	4 (2-4)	3 (2-4)	4 (3-4)	**<0.001**
Balance knee	3 (2-4)	2 (1-3)	3 (2-4)	3 (2-4)	3 (2-4)	**0.001**
**SPPB Subscore**	**Pre-op**	**Post-op d3**	**Post-op d7**	**Post-op 4-6 wk**	**Post-op 12 wk**	**p**
Gait speed total	3 (2-4)	1 (1-2)	2 (1-3)	3 (3-4)	4 (3-4)	**<0.001**
Gait speed hip	3 (1-4)	1 (1-2)	2 (1-3)	3 (2-4)	4 (3-4)	**<0.001**
Gait speed knee	3 (2-4)	1 (1-2)	2 (1-3)	3 (3-4)	4 (3-4)	**<0.001**
**SPPB Subscore**	**Pre-op**	**Post-op d3**	**Post-op d7**	**Post-op 4-6 wk**	**Post-op 12 wk**	**p**
Chair rise total	1 (1-2)	1 (0-1)	1 (0-2)	2 (1-3)	2 (1-3)	**<0.001**
Chair rise hip	1 (1-2)	1 (0-1)	1 (1-3)	2 (1-3)	2 (1-4)	**<0.001**
Chair rise knee	1 (1-3)	0 (0-1)	1 (0-2)	1 (0-3)	2 (1-3)	**<0.001**

SPPB, Short Physical Performance Battery; Md, Median; IQR, Interquartile Range; p, p-value of the Friedman Test; d, day; wk, week
